# Phenotypic and Genetic Links between Body Fat Measurements and Primary Open-Angle Glaucoma

**DOI:** 10.3390/ijms24043925

**Published:** 2023-02-15

**Authors:** Shi Song Rong, Xinting Yu

**Affiliations:** 1 Department of Ophthalmology, Massachusetts Eye and Ear, Mass General Brigham, Harvard Medical School, Boston, MA 02114, USA; 2 Department of Medicine, Brigham and Women’s Hospital, Mass General Brigham, Harvard Medical School, Boston, MA 02115, USA

**Keywords:** primary open-angle glaucoma, obesity, genetic correlation, pleiotropy analysis, genetic overlap, body mass index, waist-to-hip ratio, body fat measurements

## Abstract

The phenotypic and genetic links between body fat phenotypes and primary open-angle glaucoma (POAG) are unclear. We conducted a meta-analysis of relevant longitudinal epidemiological studies to evaluate the phenotypic link. To identify genetic links, we performed genetic correlation analysis and pleiotropy analysis of genome-wide association study summary statistics datasets of POAG, intraocular pressure (IOP), vertical cup-to-disc ratio, obesity, body mass index (BMI), and waist-to-hip ratio. In the meta-analysis, we first established that obese and underweight populations have a significantly higher risk of POAG using longitudinal data. We also discovered positive genetic correlations between POAG and BMI and obesity phenotypes. Finally, we identified over 20 genomic loci jointly associated with POAG/IOP and BMI. Among them, the genes loci *CADM2*, *RP3-335N17.2*, *RP11-793K1.1*, *RPS17P5*, and *CASC20* showed the lowest false discovery rate. These findings support the connection between body fat phenotypes and POAG. The newly identified genomic loci and genes render further functional investigation.

## 1. Introduction

Obesity is a condition characterized by excess body fat. Defined by a body mass index (BMI) of at least 30 kg/m^2^, it is one of the largest global health problems [1], is the most important risk factor for chronic disease in the United States [2], and is associated with more than 200 related complications [3], including primary open-angle glaucoma (POAG) [4,5] and increased intraocular pressure (IOP) [6]. POAG is an ocular neurodegenerative disorder and a leading cause of global irreversible blindness [7,8]. Under the direct influences of IOP, patients with POAG gradually develop retinal ganglion cell (RGC) injury, retinal nerve fiber layer thinning, characteristic optic disc cupping, and corresponding visual field defects [7,9].

The association between obesity and POAG is more sophisticated than a linear relationship. On one hand, epidemiological and clinical data support a positive association of POAG risk with obesity. Newman-Casey, Jung Y., and Chen W.D. reported from populations of European and Asian ancestries that obese patients had a 6% to 54% increase in POAG risk after multivariable adjustment [4,10,11]. Clinical evidence from two separate groups revealed a substantial decrease in IOP after successful weight management through bariatric surgery [12,13]. On the other hand, population-based studies also reported a negative or an insignificant association between obesity/body fat measurements and POAG. Na K.S. found 8% reduced risk of developing POAG in obese subjects [14], while Pasquale L.R. showed a negligible association between obesity and POAG [15]. Using BMI and waist-to-hip ratio (WHR), studies also generated a mixed picture of the POAG–obesity relationship. For the association between POAG and BMI/WHR, Jiang X. reported a positive association [16], while Pasquale L.R. and Ramdas reported a negative association [15,17]. In addition, some studies detected a significant increase in POAG risk in underweight subjects [4,14], which further complicated the picture. Therefore, efforts should be made to sift through the existing epidemiological evidence and determine the true relationship between POAG risk and body fat measurements.

Obesity is multifactorial and occurs due to complex interactions between genetics and the environment. The high heritability (*h*^2^) for different measures of obesity and body fat—BMI (*h*^2^ = 0.4–0.7) and WHR (*h*^2^~0.45)—underlines the strong effects of genetic factors in the phenotype [18]. Since 2006, significant associations of hundreds of single nucleotide polymorphisms (SNPs) with obesity, BMI, and WHR have been identified by genome-wide association studies (GWASs) [19,20]. For POAG, thus far, GWASs have mapped more than 100 gene loci that are significantly associated with POAG or IOP [21,22,23]. These discoveries have provided substantial insights into the genetic underpinnings of both disease phenotypes. These genomic data also offer a unique opportunity to the evaluation of the relationship between obesity/body fat measurements and POAG. For example, two studies have reported significant causal effects of obesity [24] and BMI [25] on POAG phenotype using two-sample Mendelian randomization methods. Therefore, further uncovering the specific gene or genomic loci that are directly responsible for the association between both phenotypes will improve the understanding of disease pathogenesis, classification and risk profiling while suggesting uncharacterized biological mechanisms.

In this study, we first confirmed the significantly increased risk of POAG in obese and underweighted populations using the existing longitudinal data and meta-analysis. We then found positive genetic correlations of POAG phenotypes with BMI and obesity phenotypes. Finally, we identified more than 20 genomic loci jointly associated with POAG and BMI. Based on epidemiological and genomic data, this study provided new evidence supporting the link between body fat phenotypes and POAG. Moreover, new target genomic loci and genes were highlighted for further functional investigations.

## 2. Results

### 2.1. Both Obesity and Underweight Increases POAG Risk in Longitudinal Epidemiology Studies

The literature search yielded 940 records. Among them, we identified 12 independent longitudinal cohorts in nine studies (Figure S1 and Table S2) [4,5,6,10,11,14,15,16,17]. Seven of the cohorts were of European ancestries [6,11,15,16,17], and five were recruited from Asian populations [4,5,10,14].

**Figure 1:**
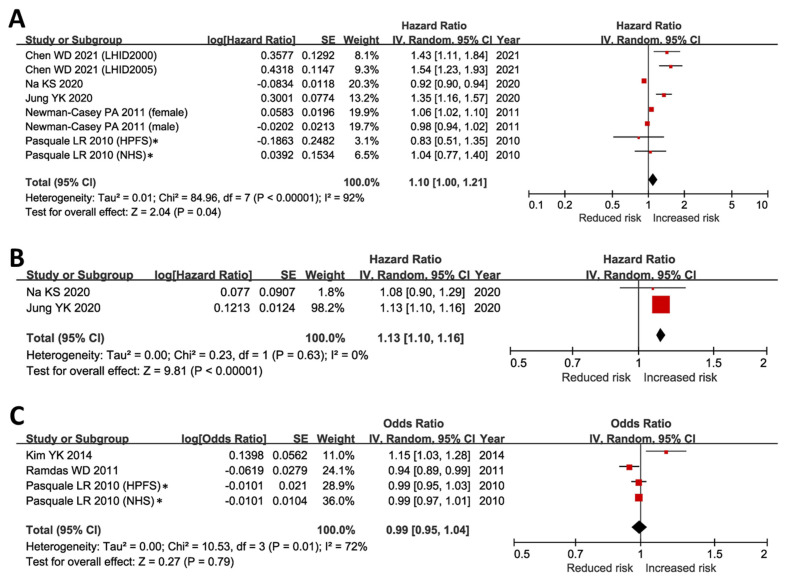
Associations of obesity, underweight, and BMI with incidence of open-angle glaucoma. (**A**) Incidence of POAG was significantly higher in obese subjects (BMI ≥ 30 kg/m^2^) when compared to individuals with normal BMI (*p* = 0.04) [4,10,11,14,15]. (**B**) Incidence of POAG was significantly higher in underweight individuals (*p* < 0.001) [4,14]. (**C**) The association of BMI with the risk of POAG was negligible when BMI was analyzed as a continuous independent variable (*p* = 0.79) [5,15,17]. BMI, body mass index; CI, confidence interval; IV, inverse variance; POAG, primary open-angle glaucoma; SE, standard error. * In Pasquale LR’s study, the incidence of POAG was <1% during the follow-up period [15]. Therefore, we included the reported relative risk in our meta-analyses of obesity and BMI outcomes, assuming that the risk ratio, odds ratio, and hazard ratio were comparable under a very low incidence of POAG [26,27]. In sensitivity analysis, the results remained unchanged after excluding the risk ratios reported in Pasquale LR’s report [15].

Association of obesity and incidence of POAG was tested in seven large cohorts (Table S2) [4,10,11,14,15]. Subsequent meta-analysis supported a significantly increased risk of developing POAG in the obese population (HR = 1.10, 95% CI 1.00–1.21, *p* = 0.03) (Figure 1A). Opposite to obesity, we further tested the associations of underweight (BMI < 18.5 kg/m^2^) with the development of POAG. In contrast to obesity, a meta-analysis of two independent cohorts from the Korean population [4,14] also linked underweight to a significantly increased risk of developing POAG (HR = 1.13, 95% CI 1.10–1.16, *p* < 0.001) (Figure 1B). These results partially explained the inconclusive correlations between BMI/WHR and POAG risk that have been reported in five large cohorts, where two were positive correlations (*p* < 0.03) [5,16], one negative correlation (*p* = 0.03) [17], and two insignificant findings [15]. Our meta-analysis of BMI’s effect on glaucoma risk, with BMI as a continuous variable, showed no significant relationship (*p* = 0.79). This refuted a simple linear link between BMI and POAG risk (Figure 1C).

All meta-analyzed studies had a Newcastle Ottawa Scale (NOS, accessed via http://www.ohri.ca/programs/clinical_epidemiology/oxford.asp, accessed on 1 December 2022) score of six and above (Table S3), suggesting a low risk of biases in the pooled outcomes. In sensitivity analysis, the pooled results remained unchanged after removing the two studies that scored six in NOS [4,14] and the relative risk/risk ratio (RR) outcomes [15].

These results suggested that risk of developing POAG may have a U-shaped correlation with BMI, which corresponded to the raised POAG risk at the lower (underweight) and the upper (obesity) end of the BMI range.

### 2.2. Genome-Wide Association Studies

In subsequent genetic analysis, we included 10 GWAS summary statistics datasets from eight GWASs, representing the target phenotypes POAG (open-angle glaucoma [28], IOP [29], and vertical cup-to-disc ratio (VCDR) [30]) and body fat measurements (obesity [31,32], BMI [33,34], and WHR [35]). GWAS summary datasets for the phenotype underweight—the lower end of the BMI measurement—were not available. Therefore, we were not able to analyze this phenotype. Features of the included GWASs and corresponding datasets are summarized in Table S4.

### 2.3. Genetic Correlation

**Figure 2:**
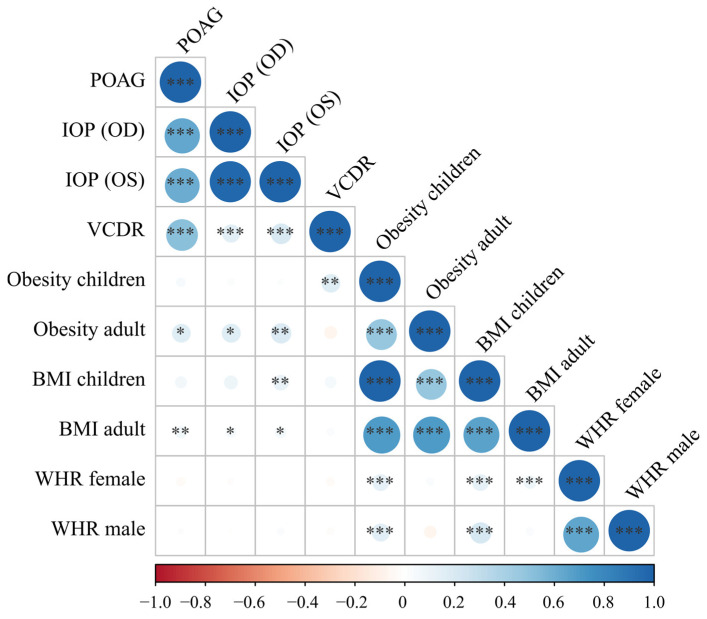
Genetic correlations of primary open-angle glaucoma-related phenotypes and body fat measurements. Obesity and BMI were positively correlated with POAG, IOP, and VCDR with suggestively significant *p* values. Glaucoma phenotypes and body fat measurements showed strong genetic correlations within their own cluster, which suggested that the genetic correlation results were valid and robust. The larger the correlation coefficient’s absolute value was, the larger the circle. The direction and value of the correlation coefficients were indicated by the color bar below the plot. BMI, body mass index; IOP, intraocular pressure (corneal-compensated, OD and OS); OD, right eye; OS, left eye; POAG, primary open-angle glaucoma; VCDR, vertical cup-to-disc ratio; WHR, waist-to-hip ratio * *p* < 0.05, ** *p* < 0.01, *** *p* < 0.001.

Linkage disequilibrium score regression was performed to evaluate the genetic correlation between POAG phenotypes and body fat measurements. We found suggestive positive genetic correlations of POAG, IOP, and VCDR with BMI and obesity, but not with BMI-adjusted WHR in either gender (*p* > 0.05) (Figure 2). The statistical significance was segregated between POAG and BMI/adult obesity (*p* values ranged from 0.03 to 0.002) (Figure 2). The average genetic correlation coefficient between adult obesity and POAG/IOP was higher than that between adult BMI and POAG/IOP (0.18 vs. 0.061; P_t-test_ = 0.0016). These results supported the existence of shared genetic factors between BMI/obesity and POAG phenotypes. Moreover, the lower correlation coefficient between adult BMI and POAG/IOP than that between POAG/IOP and obesity might be partially explained by the U-shaped correlation between POAG and BMI when both obese and underweight subjects were analyzed in the BMI GWAS datasets.

In addition, expectedly, the POAG, IOP, and VCDR phenotypes significantly correlated with each other (*p* < 0.001), and most of the body fat measurements, including BMI, obesity, and WHR, also significantly correlated with each other (Figure 2). These results supported the validity of the genetic correlation results.

### 2.4. Shared Genetic Factors between Body Fat and POAG

#### 2.4.1. Conditional Q-Q Plot

Based on the above results, we further focused on identifying the specific genetic overlaps between body fat measurements and POAG. We used the GWAS summary statistics datasets of the POAG [28], IOP (OS) [29], and adult BMI [33] phenotypes. 

**Figure 3:**
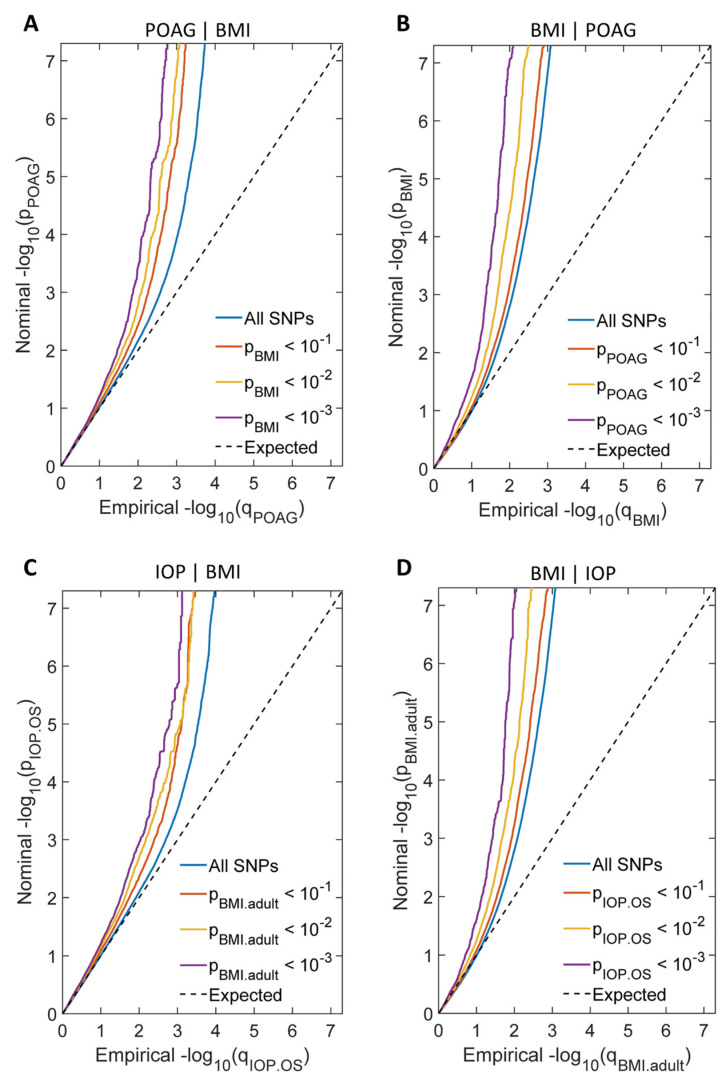
Conditional Q-Q plots showed cross-trait enrichment between POAG/IOP and BMI. (**A**) Conditional Q-Q plot of nominal—log10(p) for SNPs association with POAG as a function of statistical significance for SNPs association with BMI at the predefined stratum of *p* values. The lines with sharp leftward deviated tails showed the true associations between POAG and BMI. (**B**) Conditional Q-Q plot of nominal—log10(p) for SNPs association with BMI as a function of statistical significance for SNPs association with POAG at the predefined stratum of *p* values. The lines with sharp leftward deviated tails showed the true associations between BMI and POAG. (**C**) Conditional Q-Q plot of nominal—log10(p) for SNPs association with IOP as a function of statistical significance for SNPs association with BMI at the predefined stratum of *p* values. The lines with sharp leftward deviated tails showed the true associations between IOP and BMI. (**D**) Conditional Q-Q plot of nominal—log10(p) for SNPs association with BMI as a function of statistical significance for SNPs association with IOP at the predefined stratum of *p* values. The lines with sharp leftward deviated tails showed the true associations between BMI and IOP.

We generated a conditional Q-Q plot to visualize the cross-trait enrichment between POAG/IOP and BMI. If there was cross-trait enrichment, successive leftward deflections can be seen in conditional Q-Q plots as levels of SNP associations with the BMI phenotype increase. Our conditional Q-Q plots showed noticeable cross-trait enrichment of genetic variants between POAG/IOP and BMI (Figure 3). 

#### 2.4.2. Shared Genetic Loci between POAG/IOP and BMI

**Figure 4:**
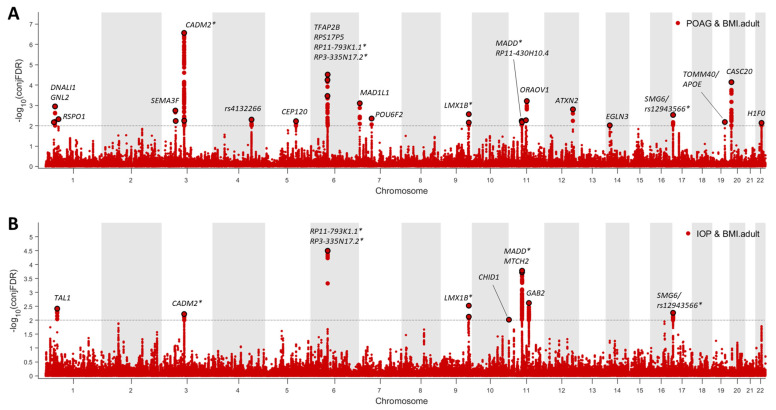
Common genetic variants jointly associated with POAG/IOP and BMI at conjFDR <0.01. (**A**). Twenty-three independent genomic loci were identified to be jointly associated with POAG and BMI. (**B**). Ten independent genomic loci were identified to be jointly associated with IOP and BMI. * There were six overlapping genomic loci that were jointly associated with POAG, IOP, and BMI. The nearby genes included *RP3-335N17.2*, *RP11-793K1.1*, *MADD*, *CADM2*, *LMX1B*, and *SMG6*.

At conjFDR <0.01, we identified 23 distinct genomic loci jointly associated with POAG and BMI (Figure 4 and Table S5). Moreover, we found 11 independent genomic loci jointly associated with IOP and BMI (Figure 4 and Table S6). Among the identified genomic loci, six showed association with POAG, IOP, and BMI (Figure 4, Tables S5 and S6). Seventeen of the identified genomic loci were not reported in previously published GWASs of glaucoma or IOP (Tables S5 and S6).

#### 2.4.3. Functional Annotations to the Identified Genomic Loci

We first obtained the expression profiles and known gene functions of the nearby genes to the independently significant SNPs (Table S7 and Figure S2). In subsequent GO enrichment analysis, none of the GO terms survived correction for multiple testing (corrected *p* values >0.05).

## 3. Discussion

In this study, we first established that obese and underweight populations have a significantly higher risk of POAG using existing longitudinal data and meta-analysis. We also discovered positive genetic correlations between POAG, BMI, and obesity phenotypes. Finally, we identified over 20 genomic loci that are jointly associated with POAG and BMI. Our study, which combines both epidemiological and genomic data, strongly supports the connection between body fat phenotypes and POAG. Additionally, we identified new genomic loci and genes that are worthy of further functional investigation.

In the GO term enrichment analysis, we did not identify a significant functional cluster from the list of potential genes prioritized by our analysis, which suggested that each genomic loci/gene could play a different role that links POAG/IOP and BMI. Noticeably, half of the prioritized genes are also expressed ubiquitously in different human tissue types, which is in line with their association with BMI, a systemic parameter, but their specific effects on the ocular condition—POAG—should be further studied. 

*ATXN2*, for example, is important for multiple cellular processes [36], including (1) repressing mTORC1 signaling pathway to limit cell size, protein synthesis, fat and glycogen utilization; (2) assisting mitochondrial autophagy and maintaining mitochondrial precursors, etc. One possibility that *ATXN2* may alter glaucoma risk is through causing mitochondrial dysfunction, because one of the significant aspects in glaucoma pathogenesis is the structural and functional impairment of mitochondria in retinal ganglion cells and their axons and synapses [37]. In addition to its role in glaucoma, ATXN2 has been linked to body fat regulation. Animal studies in mice have shown that ataxin-2 is involved in the regulation of energy metabolism including fatty acid metabolism [38]. However, more research is needed to fully understand the molecular mechanisms underlying the involvement of *ATXN2* and the other potential genes/genomic loci in body fat control and glaucoma.

There are some limitations in our study. First, the available GWAS summary statistics datasets were limited for underweight phenotype and obesity. Although we included two GWAS datasets for obesity, the sample size and statistical power of the two GWASs were incomparable to the datasets for POAG, IOP, BMI or WHR. This limited our ability to identify shared genomic loci for these two phenotypes and to test if obesity and underweight would have different shared loci with POAG. Second, in our pleiotropy analysis, we employed GWASs with potential overlapping samples, causing FDR inflation. To minimize the risk of false enrichment from population stratification or relatedness, we employed methods such as an established genomic control procedure with intergenic SNPs to control FDR inflation. Third, the discoveries were made using the GWASs conducted mainly in populations of the European ancestries. The results should be further verified in populations of other ancestral origins.

## 4. Materials and Methods

### 4.1. Meta-Analysis

We searched for original studies evaluating the association between obesity phenotypes and POAG in the PubMed database on 20 December 2022. Search strategies are shown in Table S1 [39,40]. In addition, we manually scanned the citations of relevant articles and reviews. We summarized the studies met the following criteria: (1) longitudinal study design–incident of POAG was reported; (2) evaluated the risk of developing open-angle glaucoma or its progression under exposure to obesity, different BMI levels, or WHR levels; (3) reported outcomes in odds ratio (OR), hazard ratio (HR), or relative risk (RR); (4) written in English. We excluded animal studies, case reports, reviews, abstracts, conference proceedings, editorials, and studies with incomplete data. All records were reviewed, and data were extracted/cross-checked by two reviewers (S.S.R. and X.T.Y.). We used the Newcastle–Ottawa Scale (NOS, accessed via http://www.ohri.ca/programs/clinical_epidemiology/oxford.asp, accessed on 1 December 2022) to assess the quality of the cohort studies (Appendix A) [41,42], which informed our risk of bias assessments.

Meta-analysis of longitudinal studies was conducted following our previously published methodologies [43,44,45]. Briefly, we combined study outcomes of comparable definitions using RevMan 5 (https://training.cochrane.org/online-learning/core-software/revman/, accessed on 1 December 2022). Obesity and underweight were defined as BMI ≥ 30 kg/m^2^ and BMI < 18.5 kg/m^2^, respectively. We treated each cohort as an independent record when one study reported multiple cohorts. Only fully adjusted outcomes were combined using a random-effects model—the DerSimonian and Laird method [46]. In all the cohorts, the incidence of POAG was <5% during the follow-up period. Therefore, we included relative risk or OR in our meta-analyses of obesity and BMI outcomes, assuming the relative risk, odds ratio, and hazard ratio were comparable under a very low incidence of POAG [26,27]. In sensitivity analysis, we recalculated the combined outcomes by excluding the RR results, or via leaving each cohort at a time. Heterogeneity between studies was evaluated using Q-statistic and I^2^ [47,48]. Funnel plots were generated [49,50,51].

### 4.2. GWAS Quality Control and Imputation

The study-specific design for each GWAS [28,29,30,31,32,33,34,35], including sample collection, quality control procedures, and criteria for inclusion or exclusion of SNPs, was described in the corresponding publication. In general, SNPs with call rates less than 95% and a minor allele frequency of less than 0.5% were excluded. Software used for imputation included Minimac3 and SNP2HLA, etc.

### 4.3. Genetic Correlation Analysis Using Linkage Disequilibrium Score Regressions

Linkage disequilibrium score regression (LDSR) regresses LD scores on the χ^2^ statistics of the SNPs from a GWAS to infer SNP-based heritability. Genetic correlations are estimated from the LDSR slope between two phenotypes [52]. To test genetic correlation between POAG and body fat measurements, GWAS summary statistics datasets of European ancestries for POAG, IOP [53,54], VCDR [30], obesity [31,32], BMI [33,34], and BMI-adjusted WHR [35] were curated. LD scores were computed using 1000 Genomes European data [55]. The LDSR analysis was completed using the ldsc tool kit and the following published guidelines (https://github.com/bulik/ldsc, accessed on 1 December 2022) [56].

### 4.4. Pleiotropy Analysis

In general, we performed pleiotropy analysis following established protocols and tools [57,58,59]. To reduce the risk of spurious enrichment due to population stratification or relatedness [60], we applied a genomic inflation control procedure using intergenic SNPs, which are relatively depleted of true associations [61]. This procedure was used to correct all test statistics. Moreover, we used *n* = 20 iterations of random pruning with an LD threshold r^2^ = 0.1 to define LD-blocks throughout the genome. We also excluded the major histocompatibility complex (MHC) region in the analysis due to strong SNP associations within the long-range LD region [62].

#### 4.4.1. Conditional Quantile-Quantile Plots

Conditional Q-Q plots depict the differential enrichment between pre-specified strata of SNPs. Conditional Q-Q plot was constructed by creating subsets of SNPs based on the level of association with the BMI phenotype (i.e., *p* < 0.1, *p* < 0.01, and *p* < 0.001). The data points on the Q-Q plot were weighted according to the LD structure around the corresponding SNP.

If there was no cross-trait enrichment, the nominal *p* values of POAG associations will form a straight line shown as a function of their empirical distribution. With the existence of cross-trait enrichment, successive leftward deflections can be seen in conditional Q-Q plots as levels of SNP associations with the BMI phenotype increase, i.e., *p* ≤ 1, *p* < 0.1, *p* < 0.01, and *p* < 0.001. We created Q-Q plots by considering BMI as the primary phenotype and POAG/IOP as the conditional phenotype and then by reversing the roles of the primary and conditional phenotypes.

#### 4.4.2. Conditional and Conjunction False Discovery Rates

To identify the specific pleiotropic SNPs that were significant for both POAG and body fat phenotypes, we applied conditional and conjunctional false discovery rates (FDRs) analysis (condFDR and conjFDR, respectively). FDR is a statistical method for correcting for multiple hypothesis testing and was used in pleiotropy analysis to account for the possibility of non-pleiotropy for a particular SNP [58]. To increase the power of detecting pleiotropy associated with the primary phenotype (e.g., POAG) while leveraging the association with the second phenotype (e.g., a body fat phenotype), we used the Bayesian conditional FDR method (condFDR) [57,63].

CondFDR is a modified version of FDR that takes into account the associations between genetic variants and the secondary phenotype to recalculate the P values for the primary phenotype. In our study, we used condFDR analysis to identify genetic variants associated with POAG that are dependent on the body fat phenotype. If the primary phenotype (e.g., POAG) and the second phenotype (e.g., body fat phenotype) are genetically correlated, the condFDR will rearrange the SNPs in a different order from the ranking obtained when considering the primary phenotype alone [59]. The condFDR of the primary phenotype (e.g., POAG) conditioned on body fat phenotype (POAG|body fat phenotype) and the reversing condFDR (body fat phenotype|POAG) were calculated as follows [64]:

$$cFDR\left(p_{i}|p_{j}\right)=\mathrm{Pr}(H_{0}^{\left(i\right)}|P_{i}\leq p_{i},P_{j}\leq p_{j})$$

where *p_i_* is the association of a specific SNP with the principal disease, *p_j_* is with the conditional disease. *P_i_* is the random variable of the *p* value for trait *i* among all SNPs. *H*_0_^(*i*)^ represents the null hypothesis that a specific SNP is not associated with trait *i*.

To identify pleiotropic SNPs that are jointly significant for both phenotypes, we calculated conjFDR, which is an extension of condFDR [65]. ConjFDR is defined as the larger of the two condFDR values and serves as a conservative estimate of the FDR for a pleiotropic SNP associated with both phenotypes. In our study, we used conjFDR analysis to identify shared SNPs between POAG and body fat phenotypes based on the previously calculated condFDR. The threshold for conjFDR was set at <0.01, and *p* values were corrected using the genomic inflation control procedure as described in previous studies [57,64,66]. For SNPs with multiple conjFDR values, we used the average values.

### 4.5. Functional Annotation of Shared Loci and Genes

We used FUMA [67] and DAVID [68] tools for functional annotation of the target genes. GO enrichment analysis was conducted using the PANTHER classification system [69].

## 5. Conclusions

Primary open-angle glaucoma and body fat have significant phenotypic and genetic overlaps. The shared genomic loci by POAG and body fat phenotypes render further functional investigation.

## Data Availability

We used publicly available software for the analyses and provided a list of the specific programs used in the methods section.

## Supplementary Materials

The following supporting information can be downloaded at: https://www.mdpi.com/article/10.3390/ijms24043925/s1.

## Appendix A. Newcastle–Ottawa Quality Assessment Scale for Cohort Studies

Note: A study can be awarded a maximum of one star for each numbered item within the Selection and Outcome categories. A maximum of two stars can be given for Comparability.

Selection
  (1) Representativeness of the exposed cohort
    (a) Truly representative of the average _______________ (describe) in the community *
    (b) Somewhat representative of the average ______________ in the community *
    (c) Selected group of users (e.g., nurses, volunteers)
    (d) No description of the derivation of the cohort
  (2) Selection of the nonexposed cohort
    (a) Drawn from the same community as the exposed cohort *
    (b) Drawn from a different source
    (c) No description of the derivation of the non exposed cohort
  (3) Ascertainment of exposure
    (a) Secure record (e.g., surgical records) *
    (b) Structured interview *
    (c) Written self-report
    (d) No description
  (4) Demonstration that outcome of interest was not present at start of study
    (a) Yes *
    (b) No
Comparability
  (1) Comparability of cohorts on the basis of the design or analysis
    (a) Study controls for _____________ (select the most important factor) *
    (b) Study controls for any additional factor * (these criteria could be modified to indicate specific control for a second important factor)
Outcome
  (1) Assessment of outcome
    (a) Independent blind assessment *
    (b) Record linkage *
    (c) Self-report
    (d) No description
  (2) Was follow-up long enough for outcomes to occur
    (a) Yes (select an adequate follow-up period for outcome of interest) *
    (b) No
  (3) Adequacy of follow-up of cohorts
    (a) Complete follow-up—all subjects accounted for *
    (b) Subjects lost to follow-up unlikely to introduce bias—small number lost—>____% (select an adequate %) follow-up, or description provided of those lost) *
    (c) Follow-up rate <____% (select an adequate %) and no description of those lost
    (d) No statement
