# Supplementary material for: Phenotypic and Genetic Links between Body Fat Measurements and Primary Open-Angle Glaucoma
Source: Int J Mol Sci. 2023 Feb 15;24(4):3925. doi: 10.3390/ijms24043925 (PMC9958617; doi:10.3390/ijms24043925)
Supplement: Supplementary file 1 [file ijms-24-03925-s001.zip › ijms-2168709-supplementary.pdf]

Phenotypic and Genetic Links between Body Fat Measurements and Primary Open-Angle Glaucoma

Shi Song Rong[1,*], Xinting Yu [2]

1. Department of Ophthalmology, Massachusetts Eye and Ear, Mass General Brigham, Harvard Medical School, Boston, MA 02114, USA
2. Department of medicine, Brigham and Women's Hospital, Mass General Brigham, Harvard Medical School, Boston, MA 02115, USA

* Correspondence: shisong_rong@meei.harvard.edu

Supplementary materials:

Table S1. Search strategies for the meta-analysis

Table S2. Cohort studies evaluated the association between obesity and open-angle glaucoma

Table S3. Quality assessments of included population-based cohorts

Table S4. Summary of included genome-wide association studies

Table S5. Shared pleiotropic loci between primary open-angle glaucoma and body mass index

Table S6. Shared pleiotropic loci between intraocular pressure and body mass index

Table S7. Known functions of the nearby genes

Figure S1. Literature search and results of literature review

Figure S2. Tissue expression profile of the nearby genes in GTEx v8 54 tissue types

Table S1. Search strategies for the meta-analysis

Search number	Query
1	(((("over weight"[tiab]) OR (overweight[tiab])) OR (obesity[tiab])) OR (obese[tiab])) OR (obesity[mh])
2	(("Body mass index"[mh]) OR ("body mass index"[tiab])) OR (BMI[tiab])
3	("Waist-to-Hip Ratio"[Title/Abstract]) OR ("Waist-Hip Ratio"[Title/Abstract])
4	glaucoma
5	intraocular pressure
6	IOP
7	(4 OR 5) OR 6
8	((1 OR 2) OR 3) AND 7

Table S2. Cohort studies evaluated the association between obesity and open-angle glaucoma

First author	Country	Cohort name	Sample size	Follow-up years	Measurements	Clinical outcome	Out-come	Outcome value	P*
Marshall, H. et al. (2022) [6]	Australia	PROGRESSA	355	5.28	BMI	VF progression	Beta	0.04 (0.005, 0.069)	0.013
	Canada	CLSA	23,226	nr	BMI	VCDR change	Beta	–0.007 (0.01, 0.001]	0.023
Chen, W. D. et al. (2021) [10]	Taiwan, China	LHID2000	59,695	≥3	Obesity	OAG	HR	1.43 (1.11, 1.84)	0.006
		LHID2005	60,590	≥3	Obesity	OAG	HR	1.54 (1.23, 1.94)	<0.001
Na, K. S. et al. (2020) [14]	Korea	KNHIS NHIC 2009-2015**	17,000,636	1-7 (median 5.5)	BMI<18.5	OAG	HR	1.129 (1.102, 1.157)	nr
					BMI≥30	OAG	HR	0.92 (0.899, 0.941)	nr
Jung, Y., et al. (2020) [4]	Korea	KNHIS-NSC 2002-2013**	287,553	nr	BMI<18.5	OAG	HR	1.08 (0.90, 1.29)	nr
					BMI≥30	OAG	HR	1.35 (1.16, 1.56)	nr
Kim, Y. K., et al. (2014) [5]	Korea	Gangnam Eye Study	5,021	5	BMI	OAG	OR	1.15 (1.03, 1.31)	0.029
Jiang, X., et al. (2012) [16]	USA	LALES	3,939	4	WHR (per 0.05)	OAG	OR	1.21 (1.05, 1.39)	0.007
Ramdas, W. D., et al. (2011) [17]	Netherla nds	The Rotterdam Study	3,939	9.7	BMI	OAG	HR	0.94 (0.89, 1.00)	0.03
Newman-Casey, P. A., et al. (2011) [11]	USA	i3 InVision Data Mart database	2,182,315	≥1	Obesity	OAG	HR	F: 1.06 (1.02, 1.10)<br>M: 0.98 (0.94, 1.03)	F: 0.011<br>M: 0.508
Pasquale, L. R., et al. (2010) [15]	USA	NHS	78,777	≤24	BMI≥30	OAG	RR	1.04 (0.77, 1.39)	nr
					BMI	OAG	RR	0.99 (0.97, 1.01)	0.15
		HPFS	41,352	≤24	BMI≥30	OAG	RR	0.83 (0.52, 1.35)	nr
					BMI	OAG	RR	0.99 (0.95, 1.03)	0.54

* All statistics were fully adjusted.

** The study population were recruited at completely non-overlapping time windows. Therefore, the samples were less likely to overlap and

BMI, body mass index; CLSA, The Canadian Longitudinal Study of Ageing; F, female; HPFS, The Health Professionals Follow-Up Study; HR, hazard ratio; KNHIS, Korean National Health Insurance Research Database; LALES, The Los Angeles Latino Eye Study; M, male; NHS, The Nurses' Health Study; nr, not reported; OAG, open-angle glaucoma; OR, odds ratio; PROGRESSA, Progression Risk of Glaucoma: RElevant SNPs with Significant Association; RR, rate ratio; VF, visual field; WHR, waist-to-hip ratio

Table S3. Quality assessments of included population-based cohorts

Author (Year of Publication)	Total stars	Newcastle - Ottawa Quality Assessment Scale for Population-based Cohorts*												
		Selection						Comparability		Outcome				
		1(a)	1(b)	2	3(a)	3(b)	4	1(a)	1(b)	1(a)	1(b)	2	3(a)	3(b)
Chen, W. D. et al. (2021) [10]	7		*	*	*		*	*			*	*		
Jung, Y., et al. (2020) [4]	6		*	*	*		*	*			*			
Kim, Y. K., et al. (2014) [5]	8	*		*	*		*	*		*		*	*	
Na, K. S. et al. (2020) [14]	6		*	*		*		*			*	*		
Newman-Casey, P. A., et al. (2011) [11]	8	*		*	*		*	*			*	*	*	
Pasquale, L. R., et al. (2010) [15]	8	*		*	*		*	*		*		*	*	
Ramdas, W. D., et al. (2011) [17]	8	*		*	*		*	*		*		*	*	

*Introduction and the quality assessment tool was available in Appendix A.

Table S4. Summary of included genome-wide association studies

Phenotype	First author	Year	Reported trait	Discovery cohort (ancestry/N)	Replication cohort (ancestry/N)	Significant SNPs	Reference
Glaucoma	Gharahkhani P	2021	OAG (POAG)	Cases: European / 16,677<br>Controls: European / 199,580	Cases: European/7,286; East Asian/6,935; African/3,281<br>Controls: European/107,362; East Asian/39,588; African/2,791	23	[28]
IOP	Pan-UKB team	2020	Corneal-compensated IOP: OD and OS two datasets	European / 87,054<br>Central/South Asian / 3,934<br>African / 3,291<br>East Asian / 901<br>Middle Eastern / 555<br>Admixed American / 344	nr	nr	[29,54]
VCDR	Springelkamp H	2017	VCDR	European / 21,768<br>Erasmus Rucphen / 2,131	Asian / 8,168	46	[30]
Obesity	Jiang L	2021	Adult obesity (ICD 278.1)	European / 456,348	nr	nr	[32]
	Bradfield JP	2012	Childhood obesity	Cases: European / 5,530<br>Controls: European / 8,318	Cases: European / 2,214<br>Controls: European / 2,674	5	[31]
BMI	Vogelezang S	2020	Childhood BMI	European / 39,620	nr	47	[34]
	Yengo L	2018	Adult BMI	European / 456,426	nr	941	[33]
WHR	Pulit SL	2018	BMI adjusted WHR Female and male two datasets	European (female) / 379,501<br>European (male) / 315,284	nr	Female: 258<br>Male: 84	[35]

BMI, body mass index; ICD, International Classification of Diseases; IOP, intraocular pressure (corneal-compensated, OD & OS); POAG, primary open-angle glaucoma; nr, not reported; SNP, single nucleotide polymorphisms; VCDR, vertical cup-to-disc ratio; WHR, waist-to-hip ratio

Table S5. Shared pleiotropic loci between primary open-angle glaucoma and body mass index

	SNV	Chr: Position	A1	A2	Minimum conjFDR	Nearest gene	Function	CADD score
1	rs9835904	3:85140723	A	G	2.70E-07	*CADM2**	Intronic	6.4
2	rs2579989	6:51460154	T	G	3.02E-05	*RP3-335N17.2**	Intergenic	3.3
3	rs17752199	6:51406848	G	A	5.57E-05	*RP11-793K1.1**	Intergenic	21.8
4	rs2635727	6:50820940	T	C	5.62E-05	*RPS17P5*‡	Intergenic	0.9
5	rs6054383	20:6584604	T	G	7.16E-05	*CASC20*	Intergenic	7.4
6	rs2206277	6:50798526	T	C	3.41E-04	*TFAP2B*‡	Intronic	1.0
7	rs4441044	11:69500363	G	A	6.09E-04	*ORAOV1*‡	Intergenic	0.9
8	rs4721321	7:2068470	G	A	7.84E-04	*MAD1L1*‡	Intronic	4.6
9	rs11577094	1:38026600	T	C	1.12E-03	*DNALI1*‡	Intronic	1.6
10	rs11583122	1:38053458	T	C	1.15E-03	*GNL2*‡	Intronic	15.9
11	rs12632110	3:50224225	A	G	1.79E-03	*SEMA3F*‡	Intronic	3.0
12	rs653178	12:112007756	C	T	2.50E-03	*ATXN2*	Intronic	0.3
13	rs3829849	9:129390800	T	C	2.69E-03	*LMX1B**	Intronic	11.9
14	rs12943566	17:2157774	A	G	2.94E-03	*SMG6**‡	Intronic	1.4
15	rs10248136	7:39077397	C	T	4.41E-03	*POU6F2*	Intronic	2.3
16	rs4132266	4:144086245	G	A	4.96E-03	(no nearby genes)‡	Intergenic	0.1
17	rs11038440	11:45419113	C	T	5.79E-03	*RP11-430H10.4*‡	Intronic	0.7
18	rs2303719	5:122682154	T	G	5.94E-03	*CEP120*‡	3'-UTR	6.7
19	rs10119	19:45406673	A	G	6.56E-03	*TOMM40*‡	3'-UTR / upstream	3.2
20	rs326214	11:47298360	G	A	6.71E-03	*MADD**	Exonic	19.0
21	rs2413485	22:38193920	C	T	7.23E-03	*H1F0*‡	Intergenic	5.2
22	rs12879626	14:34721134	T	G	9.54E-03	*EGLN3*	Intronic	11.2
23	rs11589571	1:38096994	C	T	9.79E-03	*RSPO1*‡	Intronic	12.2

* Genomic regions that jointly associated with primary open-angle glaucoma, intraocular pressure, and body mass index.
‡ Not reported in genome-wide association studies of glaucoma or intraocular pressure.

CADD, Combined Annotation Dependent Depletion; na, not applicable; SNV, single nucleotide variant; UTR, untranslated region

Table S6. Shared pleiotropic loci between intraocular pressure and body mass index

	SNV	Chr: Position	A1	A2	Minimum conjFDR	Nearest gene	Function	CADD score
1	rs2579989	6:51460154	T	G	3.17E-05	*RP3-335N17.2**	Intergenic	3.3
2	rs17752199	6:51406848	G	A	5.84E-05	*RP11-793K1.1**	Intergenic	21.8
3	rs10838738	11:47663049	G	A	1.66E-04	*MTCH2‡*	Intronic	4.4
4	rs326214	11:47298360	G	A	2.29E-03	*MADD**	Exonic	19.0
5	rs2450128	11:77940075	A	G	2.41E-03	*GAB2*	Intronic	0.9
6	rs3829849	9:129390800	T	C	3.01E-03	*LMX1B*	Intronic	11.9
7	rs977747	1:47684677	T	G	3.85E-03	*TAL1‡*	3'-UTR	0.6
8	rs9814516	3:85407980	T	G	6.03E-03	*CADM2**	Intronic	11.7
9	rs2235056	9:129377235	T	C	7.52E-03	*LMX1B**	Intronic	11.1
10	rs12943566	17:2157774	A	G	7.91E-03	*SMG6*‡*	Intronic	1.4
11	rs11246340	11:900809	A	G	9.63E-03	*CHID1‡*	Intronic	0.2

* Genomic regions that jointly associated with primary open-angle glaucoma, intraocular pressure, and body mass index.

‡ Not reported in genome-wide association studies of glaucoma or intraocular pressure.

CADD, Combined Annotation Dependent Depletion; SNV, single nucleotide variant; UTR, untranslated region

Table S7. Known functions of the nearby genes

Gene official symbol	Full gene name	Function of gene [68]
DNALI1	dynein axonemal light intermediate chain 1	This gene is the human homolog of the Chlamydomonas inner dynein arm gene, p28. The precise function of this gene is not known, however, it is a potential candidate for immotile cilia syndrome (ICS). Ultrastructural defects of the inner dynein arms are seen in patients with ICS. Immotile mutant strains of Chlamydomonas, a biflagellated algae, exhibit similar defects. (provided by RefSeq, Jul 2008)
GNL2	G protein nucleolar 2	Enables RNA binding activity. Predicted to be involved in ribosome biogenesis. Located in nucleolus. (provided by Alliance of Genome Resources, Apr 2022)
RSPO1	R-spondin 1	This gene encodes a secreted activator protein with two cysteine-rich, furin-like domains and one thrombospondin type 1 domain. The encoded protein is a ligand for leucine-rich repeat-containing G-protein coupled receptors (LGR proteins) and positively regulates the Wnt signaling pathway. In mice, the protein induces the rapid onset of crypt cell proliferation and increases intestinal epithelial healing, providing a protective effect against chemotherapy-induced adverse effects. Alternative splicing results in multiple transcript variants. (provided by RefSeq, Apr 2014)
TAL1	TAL bHLH transcription factor 1, erythroid differentiation factor	Enables several functions, including DNA-binding transcription factor activity; E-box binding activity; and histone deacetylase binding activity. Involved in several processes, including myeloid cell differentiation; positive regulation of cellular component organization; and positive regulation of erythrocyte differentiation. Located in chromatin and nucleoplasm. Part of transcription regulator complex. Implicated in acute lymphoblastic leukemia. (provided by Alliance of Genome Resources, Apr 2022)
CADM2	cell adhesion molecule 2	This gene encodes a member of the synaptic cell adhesion molecule 1 (SynCAM) family which belongs to the immunoglobulin (Ig) superfamily. The encoded protein has three Ig-like domains and a cytosolic protein 4.1 binding site near the C-terminus. Proteins belonging to the protein 4.1 family crosslink spectrin and interact with other cytoskeletal proteins. Multiple transcript variants encoding different isoforms have been found for this gene. (provided by RefSeq, Feb 2012)
SEMA3F	semaphorin 3F	This gene encodes a member of the semaphorin III family of secreted signaling proteins that are involved in axon guidance during neuronal development. The encoded protein contains an N-terminal Sema domain, an immunoglobulin loop and a C-terminal basic domain. This gene is expressed by the endothelial cells where it was found to act in an autocrine fashion to induce apoptosis, inhibit cell proliferation and survival, and function as an anti-tumorigenic agent. Alternative splicing results in multiple transcript variants encoding different isoforms. (provided by RefSeq, Jan 2016)

Gene official symbol	Full gene name	Function of gene [68]
CEP120	centrosomal protein 120	This gene encodes a protein that functions in the microtubule-dependent coupling of the nucleus and the centrosome. A similar protein in mouse plays a role in both interkinetic nuclear migration, which is a characteristic pattern of nuclear movement in neural progenitors, and in neural progenitor self-renewal. Mutations in this gene are predicted to result in neurogenic defects. Alternative splicing results in multiple transcript variants. (provided by RefSeq, Oct 2009)
RPS17P5	ribosomal protein S17 pseudogene 5	Unknown
TFAP2B	transcription factor AP-2 beta	This gene encodes a member of the AP-2 family of transcription factors. AP-2 proteins form homo- or hetero-dimers with other AP-2 family members and bind specific DNA sequences. They are thought to stimulate cell proliferation and suppress terminal differentiation of specific cell types during embryonic development. Specific AP-2 family members differ in their expression patterns and binding affinity for different promoters. This protein functions as both a transcriptional activator and repressor. Mutations in this gene result in autosomal dominant Char syndrome, suggesting that this gene functions in the differentiation of neural crest cell derivatives. (provided by RefSeq, Jul 2008)
MAD1L1	mitotic arrest deficient 1 like 1	MAD1L1 is a component of the mitotic spindle-assembly checkpoint that prevents the onset of anaphase until all chromosome are properly aligned at the metaphase plate. MAD1L1 functions as a homodimer and interacts with MAD2L1. MAD1L1 may play a role in cell cycle control and tumor suppression. Alternative splicing results in multiple transcript variants. (provided by RefSeq, Jan 2015)
POU6F2	POU class 6 homeobox 2	This gene encodes a member of the POU protein family characterized by the presence of a bipartite DNA binding domain, consisting of a POU-specific domain and a homeodomain, separated by a variable polylinker. The DNA binding domain may bind to DNA as monomers or as homo- and/or heterodimers, in a sequence-specific manner. The POU family members are transcriptional regulators, many of which are known to control cell type-specific differentiation pathways. This gene is a tumor suppressor involved in Wilms tumor (WT) predisposition. Alternatively spliced transcript variants encoding distinct isoforms have been found for this gene.(provided by RefSeq, Oct 2009)
LMX1B	LIM homeobox transcription factor 1 beta	This gene encodes a member of LIM-homeodomain family of proteins containing two N-terminal zinc-binding LIM domains, 1 homeodomain, and a C-terminal glutamine-rich domain. It functions as a transcription factor, and is essential for the normal development of dorsal limb structures, the glomerular basement membrane, the anterior segment of the eye, and dopaminergic and serotonergic neurons. Mutations

Gene official symbol	Full gene name	Function of gene [68]
		in this gene are associated with nail-patella syndrome. Alternatively spliced transcript variants encoding different isoforms have been found for this gene. (provided by RefSeq, Mar 2010)
CHID1	chitinase domain containing 1	Enables oligosaccharide binding activity. Involved in negative regulation of cytokine production involved in inflammatory response. Located in several cellular components, including late endosome; lysosome; and trans-Golgi network. (provided by Alliance of Genome Resources, Apr 2022)
GAB2	GRB2 associated binding protein 2	This gene is a member of the GRB2-associated binding protein (GAB) gene family. These proteins contain pleckstrin homology (PH) domain, and bind SHP2 tyrosine phosphatase and GRB2 adapter protein. They act as adapters for transmitting various signals in response to stimuli through cytokine and growth factor receptors, and T- and B-cell antigen receptors. The protein encoded by this gene is the principal activator of phosphatidylinositol-3 kinase in response to activation of the high affinity IgE receptor. Two alternatively spliced transcripts encoding different isoforms have been described for this gene. (provided by RefSeq, Nov 2009)
MADD	MAP kinase activating death domain	Tumor necrosis factor alpha (TNF-alpha) is a signaling molecule that interacts with one of two receptors on cells targeted for apoptosis. The apoptotic signal is transduced inside these cells by cytoplasmic adaptor proteins. The protein encoded by this gene is a death domain-containing adaptor protein that interacts with the death domain of TNF-alpha receptor 1 to activate mitogen-activated protein kinase (MAPK) and propagate the apoptotic signal. It is membrane-bound and expressed at a higher level in neoplastic cells than in normal cells. Several transcript variants encoding different isoforms have been described for this gene. (provided by RefSeq, Jul 2008)
MTCH2	mitochondrial carrier 2	This gene encodes a member of the SLC25 family of nuclear-encoded transporters that are localized in the inner mitochondrial membrane. Members of this superfamily are involved in many metabolic pathways and cell functions. Genome-wide association studies in human have identified single-nucleotide polymorphisms in several loci associated with obesity. This gene is one such locus, which is highly expressed in white adipose tissue and adipocytes, and thought to play a regulatory role in adipocyte differentiation and biology. Alternatively spliced transcript variants encoding different isoforms have been found for this gene. A recent study showed this gene to be an authentic stop codon readthrough target that can produce two isoforms from the same mRNA by use of alternative in-frame translation termination codons. (provided by RefSeq, Dec 2017)

Gene official symbol	Full gene name	Function of gene [68]
ATXN2	ataxin 2	This gene belongs to a group of genes that is associated with microsatellite-expansion diseases, a class of neurological and neuromuscular disorders caused by expansion of short stretches of repetitive DNA. The protein encoded by this gene has two globular domains near the N-terminus, one of which contains a clathrin-mediated trans-Golgi signal and an endoplasmic reticulum exit signal. The encoded cytoplasmic protein localizes to the endoplasmic reticulum and plasma membrane, is involved in endocytosis, and modulates mTOR signals, modifying ribosomal translation and mitochondrial function. The N-terminal region of the protein contains a polyglutamine tract of 14-31 residues that can be expanded in the pathogenic state to 32-200 residues. Intermediate length expansions of this tract increase susceptibility to amyotrophic lateral sclerosis, while long expansions of this tract result in spinocerebellar ataxia-2, an autosomal-dominantly inherited, neurodegenerative disorder. Genome-wide association studies indicate that loss-of-function mutations in this gene may be associated with susceptibility to type I diabetes, obesity and hypertension. Alternative splicing results in multiple transcript variants. (provided by RefSeq, Nov 2016)
EGLN3	egl-9 family hypoxia inducible factor 3	Enables peptidyl-proline 4-dioxygenase activity. Involved in several processes, including activation of cysteine-type endopeptidase activity involved in apoptotic process; peptidyl-proline hydroxylation to 4-hydroxy-L-proline; and response to hypoxia. Located in cytosol and nucleus. Implicated in renal cell carcinoma. Biomarker of clear cell renal cell carcinoma. (provided by Alliance of Genome Resources, Apr 2022)
SMG6	SMG6 nonsense mediated mRNA decay factor	This gene encodes a component of the telomerase ribonucleoprotein complex responsible for the replication and maintenance of chromosome ends. The encoded protein also plays a role in the nonsense-mediated mRNA decay (NMD) pathway, providing the endonuclease activity near the premature translation termination codon that is needed to initiate NMD. Alternatively spliced transcript variants encoding distinct protein isoforms have been described. (provided by RefSeq, Feb 2014)
TOMM40	translocase of outer mitochondrial membrane 40	The protein encoded by this gene is localized in the outer membrane of the mitochondria. It is the channel-forming subunit of the translocase of the mitochondrial outer membrane (TOM) complex that is essential for import of protein precursors into mitochondria. Alternatively spliced transcript variants have been found for this gene. (provided by RefSeq, Aug 2015)
CASC20	cancer susceptibility 20	Unknown
	RP3-335N17.2	Unknown
	RP11-793K1.1	Unknown

Gene official symbol	Full gene name	Function of gene [68]
	RP11-430H10.4	Unknown

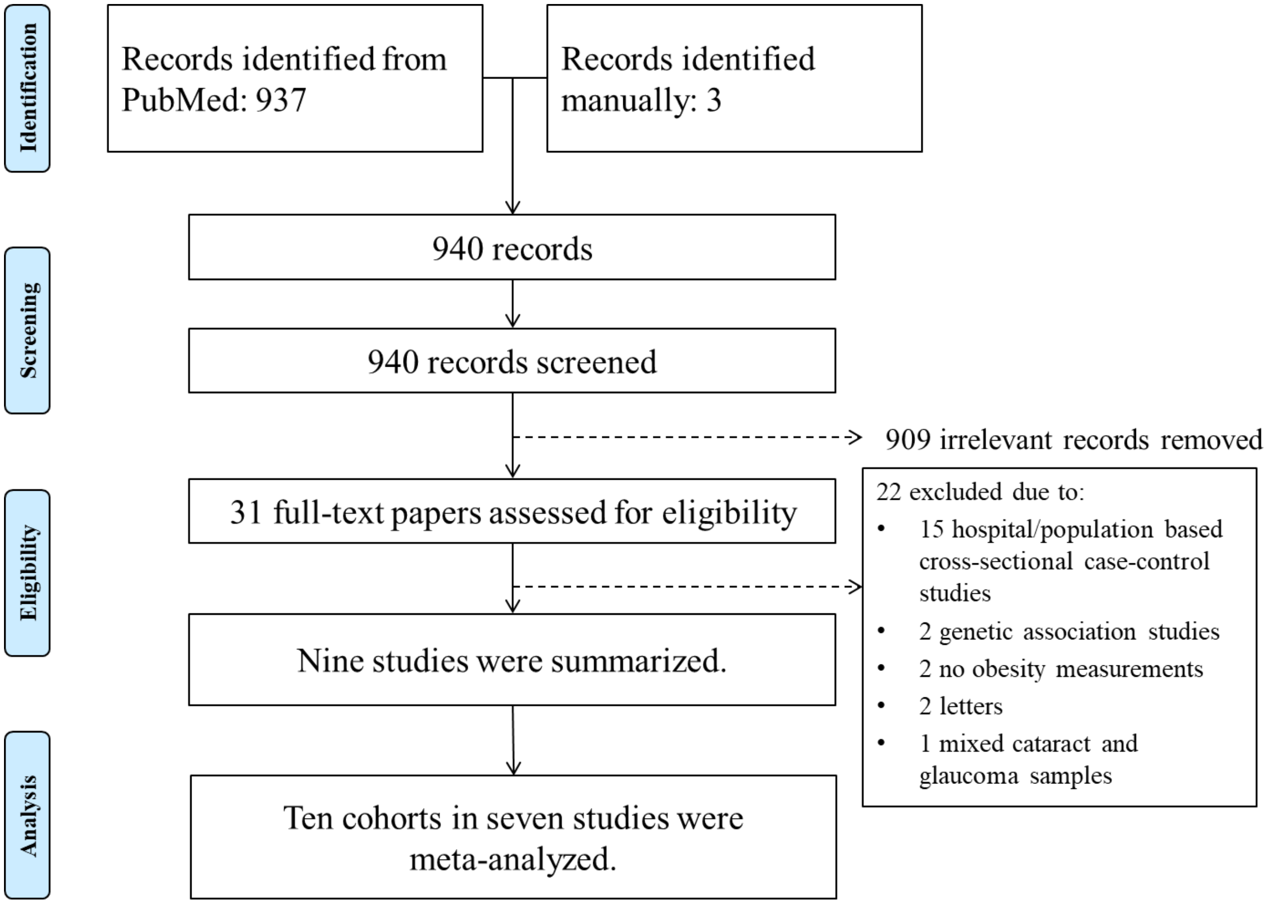

Figure S1. Literature search and results of literature review

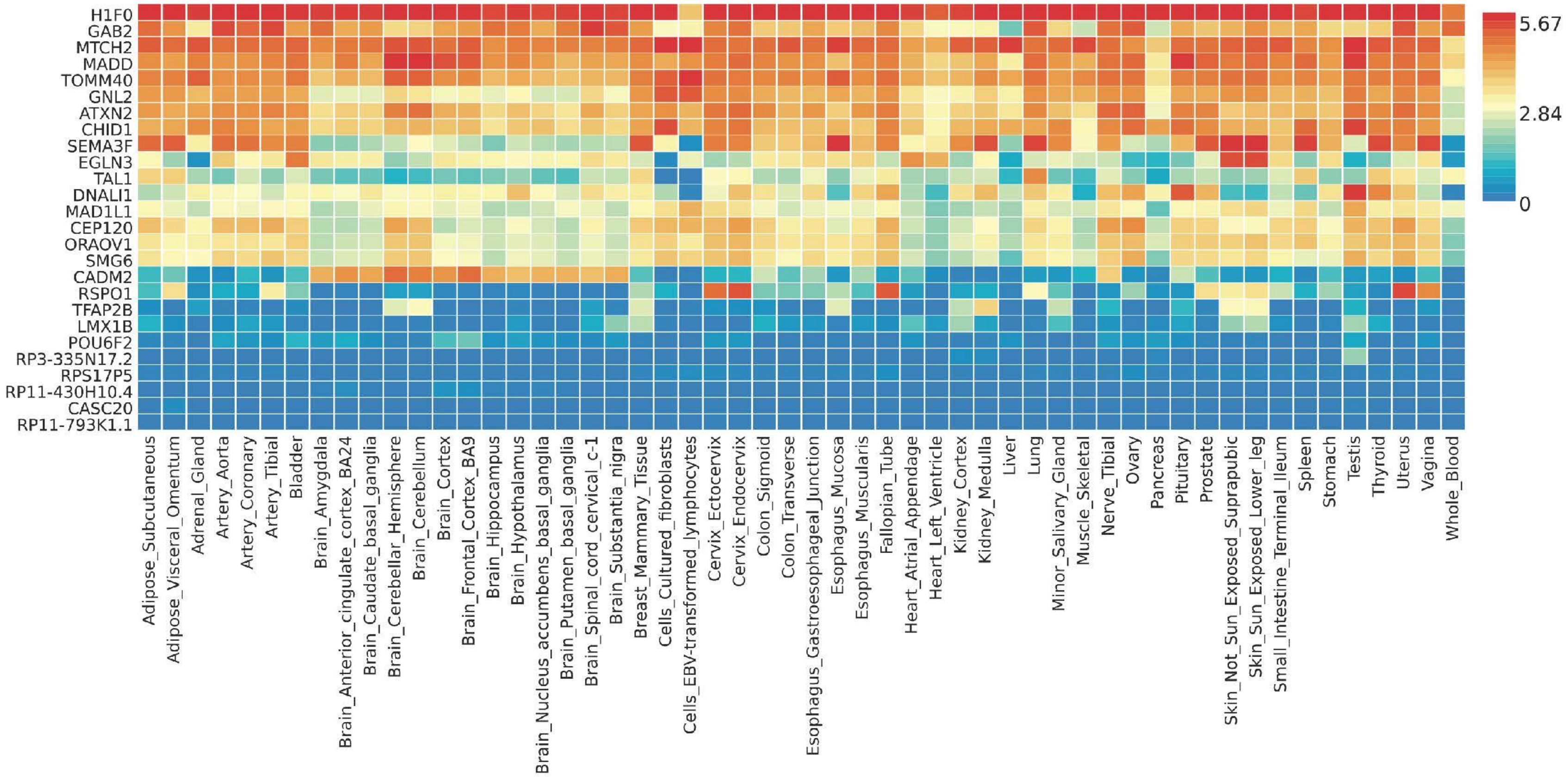

Figure S2. Tissue expression profile of the nearby genes in GTEx v8 54 tissue types
